# Concatenator, a user-friendly program to concatenate DNA sequences, implementing graphical user interfaces for MAFFT and FastTree

**DOI:** 10.1093/bioadv/vbac050

**Published:** 2022-07-21

**Authors:** Miguel Vences, Stefanos Patmanidis, Vladimir Kharchev, Susanne S Renner

**Affiliations:** Division of Evolutionary Biology, Zoological Institute, Technische Universität Braunschweig, 38106 Braunschweig, Germany; School of Electrical and Computer Engineering, National Technical University of Athens, 15780 Athens, Greece; Division of Evolutionary Biology, Zoological Institute, Technische Universität Braunschweig, 38106 Braunschweig, Germany; Department of Biology, Washington University, Saint Louis, MO 63130, USA

## Abstract

**Motivation:**

Phylogenetic and phylogenomic analyses require multi-gene input files in different formats, but there are few user-friendly programs facilitating the workflow of combining, concatenating or separating, aligning and exploring multi-gene datasets.

**Results:**

We present Concatenator, a user-friendly GUI-driven program that accepts single-marker and multi-marker DNA sequences in different input formats, including Fasta, Phylip and Nexus, and that outputs concatenated sequences as single-marker or multi-marker Fasta, interleaved nexus or Phylip files, including command files for downstream model selection in IQ-TREE. It includes the option to (re)align markers with MAFFT and produces exploratory trees with FastTree. Although tailored for medium-sized phylogenetic projects, Concatenator is able to process phylogenomic datasets of up to 30 000 markers.

**Availability and implementation:**

Concatenator is written in Python, with C extensions for MAFFT and FastTree. Compiled stand-alone executables of Concatenator for MS Windows and Mac OS along with a detailed manual can be downloaded from www.itaxotools.org; the source code is openly available on GitHub (https://github.com/iTaxoTools/ConcatenatorGui).

##  

Phylogenetic analysis of DNA sequences has become a routine task for researchers across virtually all fields of biology. In the era of high-throughput sequencing, cutting-edge research analyses genome-scale datasets with powerful algorithms (Kapli *et al.*, 2020; Patané *et al.*, 2018), often using high-performance parallel computing on local clusters or dedicated web portals (e.g. Miller *et al.*, 2010; Yu *et al.*, 2017). Such analyses are typically operated via command line interfaces that exceed the skills of many occasional users (Smith 2013). Also, for an efficient hands-on teaching of principles of systematics and evolutionary biology, user-friendly options with intuitive graphical user interfaces (GUIs) are paramount (e.g. Newman *et al.*, 2016).

One example for a community relying on user-friendly access to bioinformatics are researchers in taxonomy—the science of documenting, naming, classifying and understanding the diversity of life on Earth. Taxonomists often combine different types of data in their analyses (Padial *et al.*, 2010)—including morphological or behavioral data, but increasingly also DNA sequences (Miralles *et al.*, 2020)—yet typically are no expert bioinformaticians. For comparing homologous gene sequences, this community commonly uses GUI-driven programs, such as MEGA (Kumar *et al.*, 2018), and GUI-driven programs such as the Mesquite package (Maddison and Maddison, 2021) are also used to explore morphological data. Besides single-marker alignments, usually developed for DNA barcoding (Hebert *et al.*, 2003), taxonomists also increasingly use multiple genetic markers, either for combined analysis (typically of a concatenated matrix) or for separate analyses, for example using coalescence methods for species delimitation based on genealogical concordance. These tasks, which are also routinely applied beyond the field of taxonomy, require software programs that can combine multi-gene data into different file formats for downstream analysis (e.g. Vaidya *et al.*, 2011).

##  

Concatenator was developed in the framework of iTaxoTools, a project specifically aimed at providing a diversified and versatile set of GUI-driven bioinformatic tools to accelerate the multifaceted analyses of taxonomists (Vences et al., 2021). Concatenator is one further tool developed in this framework, conceived to ease the tasks of transforming multi-gene DNA data into different formats required as input for downstream analyses. In Concatenator, we have focused on (i) an intuitive workflow, (ii) a wide range of options for input and output of sequence data, (iii) an option to selectively (re-)align DNA sequences, (iv) optional output of guide trees calculated from single-gene and concatenated alignments and (v) implementation of options to validate output files.

##  

Concatenator follows a pipeline structure, guiding users step-by-step through the process. Input files may include one genetic marker per file (e.g. multi-file Fasta or Phylip) or several markers per file (e.g. Nexus with character set information). The program then analyses the input file(s) and provides a list of markers, number of taxa and missing nucleotides (Fig. 1). Subsequent steps include the option to delete, reorder or rename markers, align selected markers, add information on codon subsets and export the data in multiple formats, including command files for downstream model selection in IQ-TREE (Minh *et al.*, 2020). The program also can work inversely on concatenated data files, reading character set information and exporting data as packages of single-gene fasta or phylip files.

**Fig. 1.**
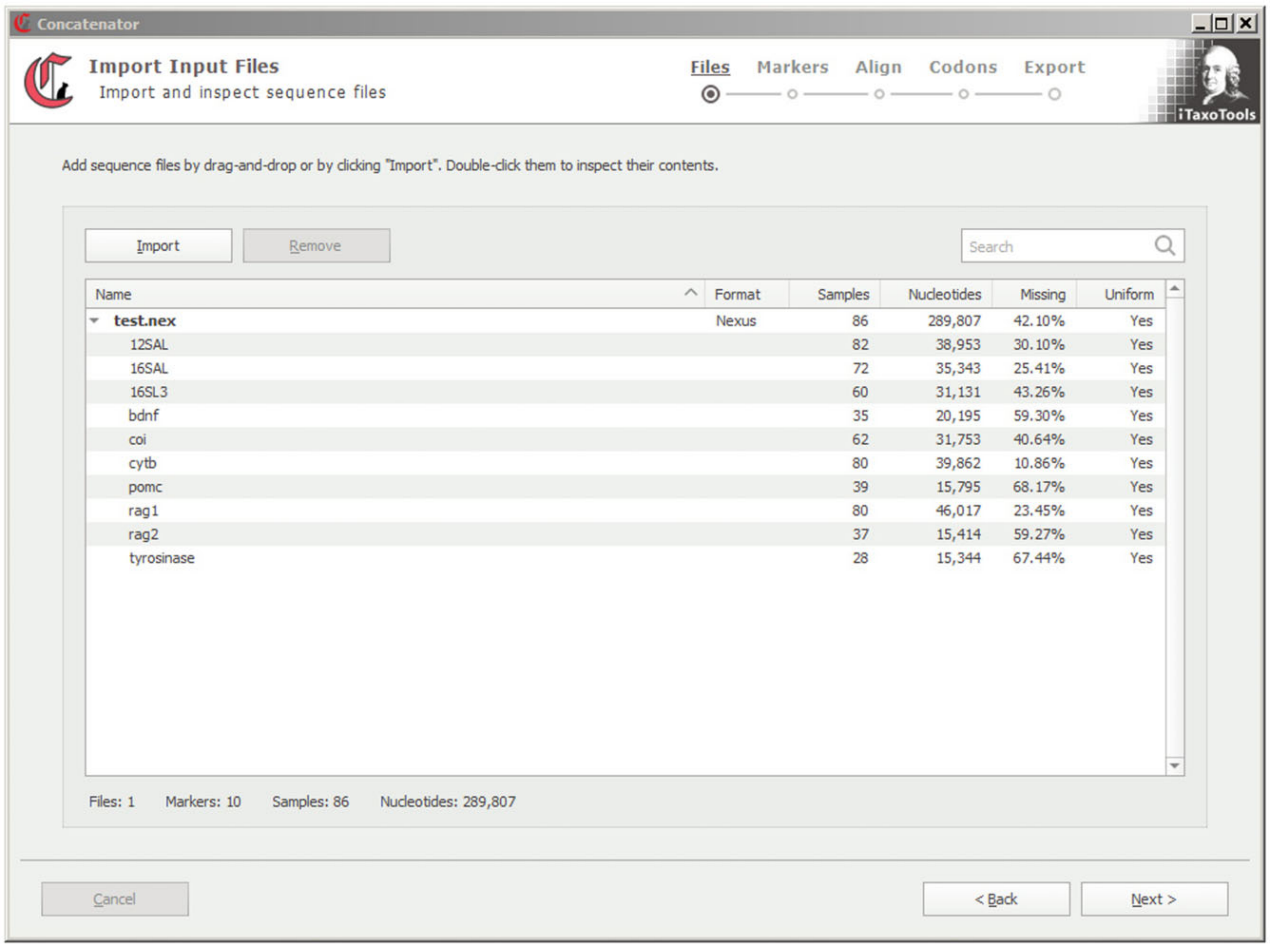
Screenshot of the starting window of Concatenator. This view is after import of a small multigene dataset from a Nexus file with character set information. Users are guided step-by-step through the different options shown in the upper right, including alignment, codon subsetting and export in different concatenated or multi-file formats

For many small- to medium-scale projects in systematics and taxonomy it is advantageous to make use of spreadsheet editors for data curation (Vences et al., 2021), enabling updates of species identity in the light of new classifications, correction of metadata and addition of new sequences. The iTaxoTools program DNAconvert supports tab-delimited spreadsheet files (tsv) for single-markers (Vences *et al.*, 2021), and Concatenator expands this option to tsv files where multiple markers are included, one per column. This input format allows inclusion of curated metadata such as specimen-voucher, clone or locality, which will then be combined to yield sequence names in the concatenated output file.

Concatenator integrates the powerful sequence alignment program MAFFT (Katoh and Standley, 2013) via a graphical user interface. The GUI version of MAFFT included in Concatenator implements a fast (FFT-NS-1) and a thorough (G-INS-i) alignment strategy for the purpose of (re-)aligning selected markers, as well as an Auto option that chooses the most appropriate of these two strategies (other alignment strategies have not been included to avoid excessive complexity of the program). In the current version, Concatenator does not include the option to automatically adjust sequences by reverse-complementing as necessary, which is included in the original MAFFT; we plan to implement this function in future versions of the program. Concatenator also offers the option to calculate exploratory single-marker and multi-marker trees under approximate maximum likelihood with FastTree (Price *et al.*, 2010). Standalone GUI tools with only MAFFT and FastTree have also been programmed and are freely distributed along with Concatenator (GitHub repositories: https://github.com/iTaxoTools/MAFFTpy**–**https://github.com/iTaxoTools/FastTreePy).

When concatenating sequences from different input files, these are identified via sequence names which for this purpose need to be identical. This process can be error-prone since already small deviations, e.g. a lack of underscores, can lead to a failed concatenation. One solution to this common issue would be fuzzy matching of names, but this is also risky as sometimes users may wish to use sequence names differing by only few characters and therefore has not been implemented in Concatenator. Curating multi-marker sequence data in spreadsheet format and then concatenating them (see above) largely avoids the risk of accidental mismatch of sequence names ins separate files but may not be practical for very large datasets. Concatenator therefore implements various options of output data validation. This includes identification of outlier sequences per marker using the SequenceBouncer algorithm (Dunn, 2020) as well as algorithms to identify cases of blocks of samples with non-overlapping sequence information. Such phenomena can occur with misspelled sample names and would hamper downstream analyses. The sensitivity of the SequenceBouncer algorithm depends on the IQR parameter (Dunn, 2020) which can be adjusted in the Concatenator GUI; as default, it is set to 20 which we found appropriate for DNA barcoding datasets of mitochondrial protein-coding genes, but it might need to be changed down to 1 for highly variable alignments, e.g. of ribosomal RNA genes of highly divergent taxa (for more details, see the Concatenator manual). Obviously, outlier detection depends on outliers being rare in the dataset and will fail if for instance, a large proportion of sequences are misaligned.

Concatenator is similar in scope to other concatenation tools such as SequenceMatrix (Vaidya *et al.*, 2011) or FASconCAT (Kück and Longo, 2014) that also focus on sequence concatenation. The option to combine sequences of different markers into a concatenated output file is also implemented in multi-purpose packages such as MEGA (Kumar *et al.*, 2018), Seaview (Gouy *et al.*, 2010) and Phylosuite (Zhang *et al.*, 2020), as well as in raxmlGUI (Edler *et al.*, 2021), while another popular phylogeny program, BEAST (Bouckaert *et al.*, 2014), combines different markers for analysis but does not output the concatenated alignment. The user-friendly alignment program Aliview (Larsson, 2014) is similar to Concatenator in implementing FastTree but lacks an explicit function for sequence concatenation. A comparison of Concatenator with six programs that also allow exporting a concatenated sequence file is provided in Table 1. Among the features unique to Concatenator are the support of tsv-formatted sequence files, batch tree inference of gene trees and concatenation tree, and seamless ‘de-concatenation’, i.e. separation of concatenated files with partition information into separate sequence files, and data validation. Options included in other programs that are not (yet) included in Concatenator are a dedicated alignment viewer, (batch) renaming of sequences/taxa, nucleotide to protein translation and model selection (see Table 1 for details).

**Table 1.** Comparison of Concatenator with six programs that also include the function of exporting a concatenated sequence file

Included features	Concatenator	SequenceMatrix	FASconCAT-G	MEGA	Phylosuite	Seaview	raxmlGUI
Graphical User Interface	Yes	Yes	No	Yes	Yes	Yes	Yes
Available for Windows	Yes	Yes	Yes	Yes	Yes	Yes	Yes
Available for Mac	Yes	Yes	Yes	Yes	Yes	Yes	Yes
Available for Linux	Yes	Yes	Yes	Yes	Yes	Yes	Yes
Main programming language	Python	Java	Perl	C++	Python	C	Electron
Installation	Standalone executable	Standalone executable (requires Java)	Uncompiled Perl code	Installer	Installer (plus Plugins)	Standalone executable (plus external files)	Installer
Drag and drop import	Yes	Yes	No	No	Yes	Yes	Yes
Import of and concatenation of multiple sequence formats	Yes	Yes	Yes	No	Yes	Yes	Yes
Support for tab-delimited sequence files and metadata	Yes	No	No	No	No	No	No
Combination of nucleotide and protein sequences	(Yes)	(Yes)	(No?)	No	(No?)	No	No
Nucleotide to protein translation	No	No	Yes	Yes		Yes	No
Selecting markers for concatenation/analysis	Yes	Yes	No	Yes	(Yes)	(Yes)	No
Selecting taxa/sequences for concatenation/analysis	No	Yes	No	(Yes)	No	No	No
Renaming of single taxa/sequences	No	Yes	Yes	(Yes)	Yes	Yes	No
‘Deconcatenation’ of alignments with partition information	Yes	Yes	No	No	No	(No)	No
Alignment of sequences	Yes (MAFFT)	No	No	Yes (Muscle/Clustal)	Yes (MAFFT/MACSE plugins)	Yes (external) (Clustal, Muscle, kalign)	No
Separate alignment by marker integrated in the concatenation process	Yes (MAFFT)	No	No	No	Yes (MAFFT/MACSE plugins)	(No)	No
Alignment viewer	No	No	No	Yes	Yes	Yes	No
Output of partition information (e.g. for IQtree, PartitionFinder, RaxML, etc.)	Yes	Yes	Yes	No	Yes	Yes	Yes
Model selection (by gene)	No	No	No	(Yes)	Yes	No	Yes
Tree inference from concatenated alignment	Yes (FastTree)	No	No	Yes (ML, MP, ME, NJ)	Yes (ML, BI plugins)	Yes	Yes (ML)
Bulk tree inference from each marker (gene trees)	Yes (FastTree)	No	No	No	Yes	No	No
Partitioning by codon position in output file	Yes	Yes	(Yes)	No	Yes	Yes	Yes
Detailed information files on input and output alignments	Yes		Yes	No	(Yes)	No	No
Identification of non-overlapping sequences	Yes	(Yes)	No	No	No	No	No
Identification of outlier sequences (per marker)	Yes (Sequence Bouncer)	No	No	No	No	No	No

While Concatenator is primarily intended for small- and medium-scale phylogenetic projects, the program is able to process large phylogenomic datasets of at least up to 30 000 markers (of alignment lengths of ∼1000 bp). For instance, concatenating a previously aligned dataset of 12 000 markers for about 80 taxa (totaling over 9 million base pairs) was successfully completed on Windows 10 and Windows 7 PCs with 3.4–3.7 GHz processors and 16 GB RAM in <4 h, and aligning 1000 markers with FFT-NS-1 took <30 min. The current implementation requires that all individual input files fit in memory.

Concatenator was written in Python version 3.8, making use of Pyside6 for the GUI and the Pandas library for data management. MAFFT and FastTree were wrapped in the form of CPython extension modules. Concatenator and all related modules are available as installable Setuptools packages and expose their functionality through simple Python APIs. Standalone executables for Windows and Apple Macintosh (running both with Intel and Apple Silicon processors) have been produced using Pyinstaller. The backend uses an extensible modular design in which configurable protocols are defined for reading/writing each file format and feeding to/from a standardized stream of markers. This consolidates the inherently different formats and allows for data analysis and manipulation. The GUI takes advantage of this design to display simple analytics and to enforce format-specific modifications on demand.

We envisage future distributions of Concatenator to include improved support for protein sequences as well as morphological matrices, translation of DNA into protein sequences and GUI access to full functions of MAFFT and FastTree.

## Data Availability

The data underlying this article are available in the following repositories: The source code is openly available (GPL 3.0 license) on the GitHub repository (https://github.com/iTaxoTools/ConcatenatorGui). Compiled stand-alone executables of Concatenator for MS Windows and Mac OS along with a detailed manual are available under from Github under https://github.com/iTaxoTools/ConcatenatorGui/releases/tag/v0.2.1, as well as from www.itaxotools.org. Standalone GUI versions of MAFFT and FastTree are available from https://github.com/iTaxoTools/MAFFTpy and https://github.com/iTaxoTools/FastTreePy).
